# UFO-Net: A Linear Attention-Based Network for Point Cloud Classification

**DOI:** 10.3390/s23125512

**Published:** 2023-06-12

**Authors:** Sheng He, Peiyao Guo, Zeyu Tang, Dongxin Guo, Lingyu Wan, Huilu Yao

**Affiliations:** 1 School of Physical Science & Technology, Guangxi University, Nanning 530004, China; 2 School of Electrical Engineering, Guangxi University, Nanning 530004, China; 3 Faculty of Science and Technology, Beijing Normal University-Hong Kong Baptist University United International College, Zhuhai 519087, China

**Keywords:** point cloud, classification, augmented sampling and grouping, transformer-based, UFO attention

## Abstract

Three-dimensional point cloud classification tasks have been a hot topic in recent years. Most existing point cloud processing frameworks lack context-aware features due to the deficiency of sufficient local feature extraction information. Therefore, we designed an augmented sampling and grouping module to efficiently obtain fine-grained features from the original point cloud. In particular, this method strengthens the domain near each centroid and makes reasonable use of the local mean and global standard deviation to extract point cloud’s local and global features. In addition to this, inspired by the transformer structure UFO-ViT in 2D vision tasks, we first tried to use a linearly normalized attention mechanism in point cloud processing tasks, investigating a novel transformer-based point cloud classification architecture UFO-Net. An effective local feature learning module was adopted as a bridging technique to connect different feature extraction modules. Importantly, UFO-Net employs multiple stacked blocks to better capture feature representation of the point cloud. Extensive ablation experiments on public datasets show that this method outperforms other state-of-the-art methods. For instance, our network performed with 93.7% overall accuracy on the ModelNet40 dataset, which is 0.5% higher than PCT. Our network also achieved 83.8% overall accuracy on the ScanObjectNN dataset, which is 3.8% better than PCT.

## 1. Introduction

As sensors become more prevalent in capturing geometric information in 3D scenes, point cloud classification has become increasingly significant for various graphical and visual tasks. Point clouds, as physical 3D world data or an electronic signal, are widely used in mapping, autonomous driving, remote sensing, robotics, and metadata [1,2,3,4,5]. Point cloud data are usually generated by optical sensors, acoustic sensors, LiDAR (light detection and ranging), and other direct or indirect contact scanners [3,6]. Specifically, researchers can obtain feature information through convolutional neural networks (CNNs) [7,8], which can then be used in subsequent processing tasks. Unlike 2D images, 3D point cloud data are nonuniform and unstructured. Point cloud processing can yield rich spatial location features and texture geometry information by designing different algorithms [9,10,11,12] to complete some 3D tasks. Point cloud classification plays an important role in many fields, such as object recognition in machines. The focus of this paper is on the shape classification of the point cloud, which is a significant task for point cloud processing.

Aimed at the existing works on classification tasks, several properties can be summarized as the following aspects. Applicability. Three-dimensional applications rely on classification tasks. In order to accurately identify the ground objects, researchers design a large number of networks to improve the score of point cloud tasks from the underlying theory. For example, robot fetching technology and face recognition technology require mature classification schemes to be successfully applied. Complexity. For the design of a brand-new method to solve hard-core tasks, simple point networks cannot complete the complex demands. Almost all models with hierarchical structures require complex array operations. Point cloud classification still requires researchers’ efforts to continue to promote theoretical analysis and model building.

Different from convolutional neural networks that process natural language and image data, CNNs cannot be applied to unordered 3D point cloud data directly. Therefore, training neural networks on point sets is a challenging task. The success of CNNs in point cloud analysis has attracted considerable attention. The rapid development of deep learning facilitates the diversification of methods for point cloud processing tasks. After recent years of research, point cloud processing methods have been derived based on a grid format or on converting them to multi-view images. All these point cloud processing methods give good results in point cloud classification tasks. However, numerous experiments have shown that these transformations lead to large computational demands and even loss of much geometric information. The point-based approaches effectively alleviate this aspect of deficiency. PointNet [10] is a pioneering point-based approach. It obtains the features of the input point cloud by directly processing each point using shared MLPs and a max-pooling symmetry function. This method learns the relative relationships between points by designing models to resolve the irregularities of the point cloud. However, PointNet ignores the correlation features of points in local areas due to the direct processing of points to obtain shape-level features, which leads to an imbalance between local domain and global features. PointNet++ [11] proposes a hierarchical approach to extracting local features, and the results showed the importance of local features in point cloud analysis. To further investigate the extraction of local features, DGCNN [12] is a new scheme to explore local features. DGCNN not only refers to the previous work but proposes a unified operator to obtain local features. Although PointNet++ and DGCNN consider mining local region features, almost all of them use a max-pooling strategy to aggregate features. This single operation considers only the most prominent feature, ignoring the other relevant geometric information. Consequently, the local information of the point cloud is not fully exploited. Therefore, to further ameliorate the performance and generalization capability, we introduce a lightweight local geometric affine module. This approach addresses point sparsity and irregular geometric structures in the local threshold.

Recently, with the strong expressiveness of the transformer structure in the field of natural language processing and image recognition, attention mechanisms have also been widely used in point cloud learning tasks. Since transformers are permutation invariant, they are well suited for point cloud learning [13,14,15,16]. The original structural components of the transformers are mainly composed of input encoding, position encoding, and self-attention (SA) mechanisms [17]. The attention mechanism is the core structure of the transformer. In detail, the attention mechanism takes the sum of the input encoding and the position encoding as input. Attention weights are obtained by dot-producing queries and keys. Therefore, the attention feature is a weighted sum of all values with attention weights. It is due to the nature of this obtained correlation between features that the extraction of point location features seems to be very effective. Naturally, the output of attention represents the features of the input sequence, which can be learned by the subsequent multi-layer perceptrons to complete the point cloud analysis. In summary, inspired by UFO-ViT [18], we propose a new framework, UFO-Net, adopting the idea of a unified force operation (UFO) layer that uses the $L_{2}$-norm to normalize the feature map in the attention mechanism. UFO decomposes the transformation layer into a product of multiple heads and feature dimensions. The point cloud features are then obtained by matrix multiplication.

The main contributions of the proposed model are the following three aspects:

(1) A novel network UFO-Net conforming to the point cloud input, which leverages the stacked UFO layers to replace the original attention mechanism in PCT [16]. UFO incorporates the softmax-like scheme CNorm, which is a novel constraint scheme. The essence of CNorm is a common $L_{2}$-norm. CNorm learns point-to-point relational features by generating a unit hypersphere [18]. Furthermore, the offset matrices [16] introduced in UFO attention are effective in reducing the impact of noise and providing sufficient characteristic information for downstream tasks.
(2) We observe that the input coordinates of the point cloud are less correlated with the features, and while the attention mechanism learns global features effectively, it tends to overlook some local features. Thus, we introduce an augmented sampling and grouping (ASG) module that rethinks the sampling and grouping of the point cloud to improve association between points. ASG selects different points by introducing the farthest point sampling (FPS) algorithm and the k-nearest neighbor (KNN) search. A lightweight geometric affine function is used to solve uneven sampling.
(3) We perform extensive experiments and analyses on two publicly available benchmark datasets—ModelNet40 and ScanObjectNN. Results verify the effectiveness of our framework, which can achieve competitive scores compared to state-of-the-art (SOTA) methods. The proposed framework provides a promising approach for point cloud tasks.

## 2. Related Works

Traditional 3D data algorithms are commonly used in the fields of robot vision navigation, artificial verification, and 3D reconstruction [1,2,8]. Recently, convolutional neural networks (CNNs) have facilitated the development of image recognition due to their robustness advantage in feature extraction [5]. Gradually, these approaches have been applied to the 3D domain, deriving intelligent point cloud processing schemes. These methods provide some practical neural networks to process point cloud data directly or indirectly and successfully mine the potential features of point cloud data. To some extent, the ability to extract features can determine the accuracy of point cloud classification tasks. To address these challenges, many researchers have designed different neural networks to implement point cloud tasks by modifying the input form of point clouds. In this section, we broadly review the existing approaches, which are generally subdivided into four aspects overall. 

### 2.1. Voxel-Based Methods

Truc Le et al. [19] first adopted a volumetric grid structure to voxelize the unsorted 3D point clouds into a regular 3D grid structure. This approach is a 3D convolutional mesh dealing with a constant number of points and allows better learning of local geometric features by using approximation functions. For example, VoxNet [20] pioneers the implementation of 3D learning. However, these methods have difficulty acquiring high-dimensional features due to the rasterization process, causing a large amount of memory consumption and requiring complex computational efforts. Clearly, these methods also have difficulty capturing high-resolution or fine-grained features due to sparsity. Furthermore, in order to alleviate the problem of large memory consumption and storage difficulties, Octree-based [21] and Kd-tree-based [22] methods refine the model’s performance. Vv-Net [23] proposes a variational autoencoder (VAE) and radial basis function (RBF) to represent voxels, which allow the model to further learns local features. Park et al. [13] proposed a lightweight self-attention module to encode the voxel hash architecture for efficiency. The voxel-based methods provide a new strategy for calculating the unstructured 3D point cloud.

### 2.2. Graph-Based Methods

Graph-based neural networks are gradually being studied and applied to irregular point cloud data, and this innovation increases the diversity of point cloud learning tasks. Graph convolutional neural networks (GNNs) are often used to learn local geometric features between multiple points. For example, the pioneered edge-conditional convolution (ECC) [24] converts the disordered point cloud data into graphs. Jing et al. [25] proposed a novel feature extraction module based on an attention-pooling strategy called the attention graph module (AGM), which constructs a topology structure in the local region and aggregates the important features using the novel and effective attention-pooling operation. The 3DGCN [26] introduces a learnable 3D kernel structure to guarantee scale invariance and a 3D graph max-pooling operator to obtain more features. In addition, to make spectral convolution kernels applicable to different graph topologies, Lin et al. [27] proposed SPH3D-GCN, a separable spherical convolutional layer for effective graph neural networks. DeepGCNs [28] adopts a deep graph convolutional network that can capture local and global features of point clouds by transferring concepts such as residual/dense connections and dilated convolutions from CNNs to GCNs. Ulteriorly, The GAPNet [29] designing graph attention point layer (GAP layer) learns point features by assigning different attention weights to the neighborhoods, which enhances the robustness of the network. The graph-based methods are flexible in processing irregular dates, achieving competitive performance on point cloud learning.

### 2.3. Point-Based Methods

The PointNet [10] network is a well-known point-based approach that learns point-wise features using multilayer perceptrons (MLPs) without data preprocessing. Then, it uses a channel-wise symmetric function (max-pooling) to obtain global feature information. However, PointNet lacks the ability to extract more fine-grained structures and local features. PointNet++ [11] fills this deficiency by designing two hierarchical schemes for local feature extraction. The hierarchical structure consisted of set abstraction modules. Each set abstraction module consists of a sampling layer, a grouping layer, and a mini-PointNet layer. The set abstract scheme improves feature aggregation and enhances the performance of PointNet. Experiments with PointNet and PointNet++ show that point-based approaches can learn point features more effectively in point cloud learning. In addition, Xu et al. [30] proposed GS-Net to effectively learn point descriptors with holistic context to enhance the robustness to geometric transformations. Li et al. [31] proposed an X-conv operator that combined geometric and color features of nearby points to compute convolutional filters. Overall, point-based approaches offer solutions for many scholars to study point clouds.

### 2.4. Attention-Based Methods

The attention mechanism originates from natural language processing [17,32,33]. Tao et al. [34] improved the attention mechanism by proposing multi-head attention and solely relying on attention itself. Compared to the limited receptive fields of CNNs, a transformer characterized by the attention mechanism shows its capabilities in feature capturing [13,16]. Several attention-based methods for point cloud classification have been proposed recently. For example, Zhao et al. [15] considered the self-attention operator and position encoding to act on the neighborhood near each point, leading to a network purely based on self-attention and point-wise manipulations. The point cloud transformer (PCT) network proposed by Guo et al. [16] enhances the input embedding with the support of farthest point sampling and nearest neighbor search. PCT also modifies the self-attention mechanism and proposed offset attention to better complete the point cloud tasks. Additionally, SA-Net [35] proposes the skip attention mechanism to fuse local region features from the encoder to the point features of the decoder, which selectively conveys geometric information at different resolutions. Han et al. [36] proposed an end-to-end architecture, dubbed the Cross-Level Cross-Scale Cross-Attention Network (3CROSSNet), to extract features from different scales or resolutions. To better obtain the features of different scales, CSANet [37] proposes a cross self-attention network and a multi-scale fusion module to adaptively consider the information of different scales and establishes a fast-descent branch to bring richer gradient information. Additionally, Qiu et al. [38] adopted the idea of error correction feedback structure to fully capture the local features of point clouds, leading to a geometric back-projection network for point cloud classification.

## 3. Materials and Methods

In this section, we illustrate how UFO-Net can be used for point cloud classification tasks. The designed details of UFO-Net are also systematically presented as follows.

**Figure 1:**
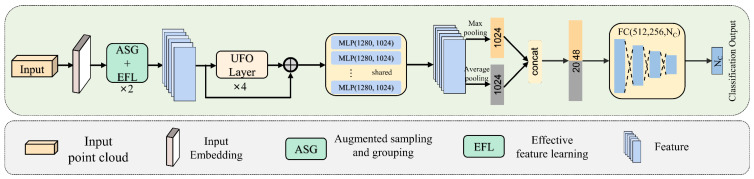
The architecture of UFO-Net.

The overall architecture of UFO-Net is depicted in Figure 1. It consists of four main components: (1) backbone: a backbone for mining features from point clouds; (2) augmented sampling and grouping (ASG) modules: two ASG modules designed to extract features from different dimensions; (3) stacked UFO attention layers: four stacked UFO attention layers to extract more detailed information and form the global feature; (4) prediction heads: global feature classified by the decoder. In detail, UFO-Net aims to transform the input points into a new higher dimensional feature space, which can describe the affinities between points as a basis for various point cloud processing tasks. Mapping points to high-dimensional space enhances the extraction of local and global features of the point cloud. The encoder of UFO-Net starts by embedding the input coordinates into a new feature space. The embedded features are later fed into two cascaded ASG and EFL modules to obtain more local detailed information. The detailed features are then fed into 4 stacked UFO layers to learn a semantically rich and discriminative representation for each point, followed by a linear layer to generate the output feature. Overall, the encoder of UFO-Net shares almost the same philosophy of design as PCT. We refer the reader to [16] for details of the original point cloud transformer.

### 3.1. Augmented Sampling and Grouping Module

In 3D point cloud operations, the neighborhood of a single point is defined by the metric distance in the 3D coordinate system. Due to the sparse local regions and irregular geometric structure of the point cloud, the sampling and grouping [16] operation cannot capture the different 3D geometric structure features among different regions effectively. This indirectly leads to a learning bottleneck in the subsequent nonlinear mapping layer, and a different extractor is required.

This paper uses a KNN search scheme based on comparative experiments. However, the existing local shape model with KNN is vulnerable to the local density of the point cloud. In other words, points near the centroid are feature-rich, while points far from the centroid are easily ignored. Therefore, we seek an optimized feature extractor. We draw upon the ideas of PCT [16] and PointNorm [39] to design an augmented local neighbor aggregation strategy ASG.

The ASG module introduces a geometric affine operator to local feature extraction. First, the input coordinates are projected using two MLPs to increase the dimension to $C_{in}$. In this paper, $C_{in}$ is taken as 64. Then, the specific implementation of the ASG module is divided into three steps: (i) selecting local centroids by using FPS; (ii) obtaining grouped local neighbors (GLNs) using KNN based on Euclidean distance; (iii) normalizing the GLNs by using the affine function. To obtain features from different local regions, the GLNs are passed through a lightweight geometric affine function. This operation can help overcome the disadvantage of uneven sampling.

The feature process of ASG can be simply described as follows:

(1) $$P_{knn}{=cat(P}_{knn}{,\;xyz}_{knn})$$

(2) $$F_{C}{=cat(P}_{C}{,\;xyz}_{C})$$

(3) $$F_{L}=\alpha \;⨀\;\frac{F_{knn}-\;I\left(F_{C}\right)}{\sigma +\varepsilon }+\beta$$

(4) $$P_{ASG}{=cat(F}_{L}{,\;P}_{C})$$

**Figure 2:**
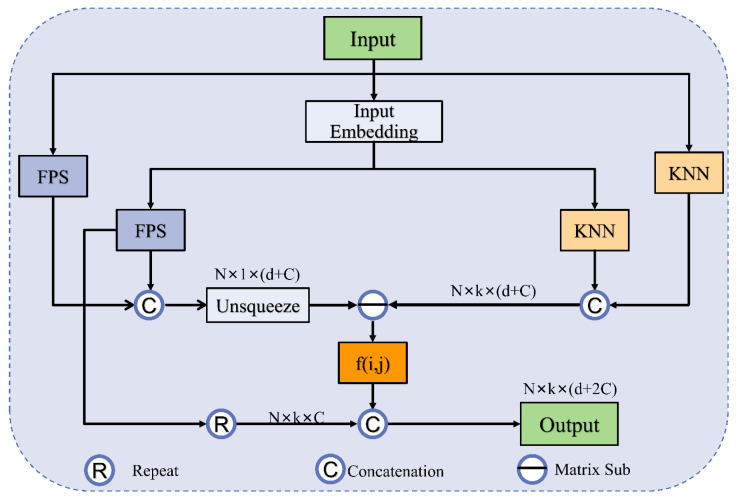
The illustration of the augmented sampling and grouping (ASG) module.

The ASG process is shown in Figure 2, where $P_{knn}$ is the *k* neighbor features found by KNN from the projection coordinates, $P_{C}$ is the *k* neighbor features found by FPS from the original coordinates, ${xyz}_{knn}$ is the *k* neighbor points found by KNN from the original coordinates, and ${xyz}_{C}$ is the centroid computed by the FPS algorithm from the original coordinates. In addition, $\alpha \;\in {\;R}^{d}$ and $\beta \;\in {\;R}^{d}$ are learnable affine transformation parameters, ⨀ denotes the Hadamard production of element directions, and ε is a number $10^{-5}$ that keeps the value stable. *I* is the unsqueeze operation, and *f* (*i, j*) is denoted as the lightweight geometric affine function $F_{L}$. Very importantly, σ is a scalar describing the deviation of features between all local groups and channels. It is this method that transforms the local features into a normally distributed process that maintains the geometric properties of the original points. Specifically, this method enhances the identification of domain features in the vicinity of each centroid. The sizes of the point cloud are decreased to 512 and 256 points within the two ASG layers.

Herein, differently from the sampling and grouping method, ASG continues to consider the projection features sampled at the farthest point. The input feature is the matrix $N_{out}{\;\times \;(d+2C}_{in})$ with $N_{out}$ subsampling points, *d*-dim coordinates, and two $C_{in}$-dim projection features. The output is the feature matrix $N_{out}{\;\times \;k\;\times \;(d+2C}_{in})$, where *k* is the number of points in the nearest domain of the centroid.

### 3.2. Effective Local Feature Learning Module

**Figure 3:**
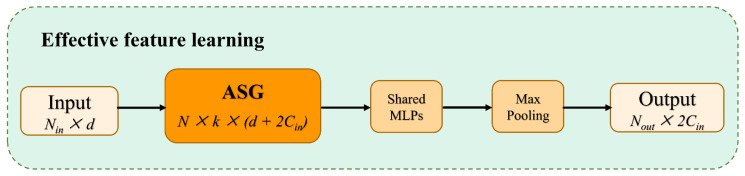
The architecture of effective feature learning (EFL).

The existing works [10,40,41] usually use symmetric functions, such as max/mean/sum pooling, to downscale and preserve the main features to solve the disorder of point clouds. The features obtained from the original point clouds processed by the ASG module lack global properties. In order to utilize the feature information collected from ASG, it is necessary to find a reasonable bridging technique between the two feature processing methods, the ASG module, and the following UFO layers. To solve this problem, an effective local feature learning (EFL) module is designed as Figure 3. Usually, the max-pooling function is applied to *k* neighbors of each elaborated local graph to obtain feature representations that aggregate local contexts as the center. Here, we denote the EFL module as:

(5) $$F_{EFL}{=M(A(A(P}_{ASG})))$$

For local sampling and grouping regions, $P_{ASG}$—a shared neural network comprising two cascaded LBRs *A* and a max-pooling operator *M* as symmetric functions—is used to aggregate features. The alignment invariance of the point cloud can be fully guaranteed by EFL. By this learning, the output size of EFL changes from the input matrix $N_{out}{\;\times \;k\;\times \;(d+2C}_{in})$ to the feature size $N_{out}{\;\times \;2C}_{in}$.

### 3.3. Stacked UFO Attention Layers

To develop the exposition of the single UFO attention layer, we first revisit the principle of the self-attention (SA) mechanism. The key to the SA mechanism is made up of query, key, and value matrices, which are denoted by *Q*, *K*, and *V*, respectively. The *Q*, *K*, and *V* matrices are generated from encoded local features using linear transformations [33]. Here, $d_{k}$ is the dimension of the key vectors, and the *softmax* function is applied to the dot product of the query and key matrices. Formally, the traditional SA mechanism is expressed as:

(6) $$Attention(Q,\;K,\;V)=softmax(\frac{{QK}^{T}}{\sqrt{d_{k}}})V$$

**Figure 4:**
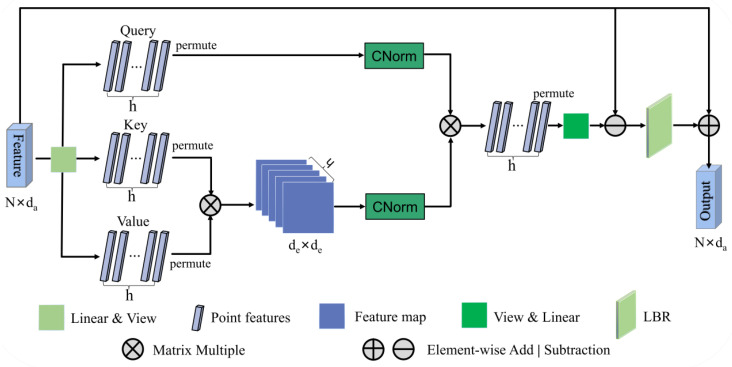
Description of the single UFO attention layer.

The UFO attention mechanism is explained in the forthcoming note. The architecture of the single UFO attention layer is depicted in Figure 4. We use linear transformation and view operation to convert the input features $F_{EFL}$ into three new representations: query, key, and value matrices. Given the input feature mapping $F_{EFL}\;\in {\;R}^{{N\times d}_{a}}$, *N* is the number of point clouds and $d_{a}$ is the feature dimension. Formally, the feature dimension $d_{a}{=h\;\times \;d}_{e}$, where *h* is the number of head and *d_e_* is the dimension of each head.

Then the single UFO attention layer is expressed as:

(7) $${U(x)=CN(Q}_{U}{)\cdot CN(K}_{U}^{T}V_{U})\;$$

(8) $$Q_{U}{=\psi (F}_{EFL})\;\in {\;R}^{{N\times h\times d}_{e}}$$

(9) $$K_{U}{=\Phi (F}_{EFL})\;\in {\;R}^{{N\times h\times d}_{e}}$$

(10) $${\;V}_{U\;}{=\gamma (F}_{EFL})\;\in {\;R}^{{N\times h\times d}_{e}}$$

where *ψ*, *Φ*, and *γ* are linear transformation and view operation. After permutation, $Q_{U}\;\in {\;R}^{{h\times N\times d}_{e}}$*,* $K_{U}^{T}\;\in {\;R}^{{h\times d}_{e}\times N}$*,* $V_{U}\;\in {\;R}^{{h\times N\times d}_{e}}$. Note that *h* = 4 was selected by the ablation experiment. We compute the product of $K_{U}^{T}$ and $V_{U}$ to obtain the spatial correlation matrix *KV_Attention* for all points. Next, we use CN to normalize *KV_Attention* to obtain *KV_Norm*. At the same time, we use CN to normalize *Q_U_* to obtain *Q_Norm*. It is a common *L*_2_-norm, but it is applied along two dimensions: the spatial dimension of $K_{U}^{T}V_{U}$ and the channel dimension of *Q_U_*. Thus, it is called CrossNorm. Then, permutation and view operation are also adopted.

CrossNorm (CN) is computed as follows:

(11) $$\mathrm{CN}\left(x\right)=\frac{\lambda x}{\sqrt{\sum_{i=0}^{h}x^{2}}}$$

where *λ* is a learnable parameter initialized as a random matrix and *x* is the transformed feature. This generates *h* clusters through the linear kernel method. The operation process can be described as:

Let $q_{i}=CN\left[\left({\left[Q_{U}\right]}_{i0},{\left[Q_{U}\right]}_{i1},\ldots ,{\left[Q_{U}\right]}_{ih}\right)\right]{,\;k}_{i}=CN\left[\left({\left[K_{U}^{T}V_{U}\right]}_{0i},{\left[K_{U}^{T}V_{U}\right]}_{1i},\ldots ,{\left[K_{U}^{T}V_{U}\right]}_{hi}\right)\right]$. Then, U(*x*) can be represented as:

(12) $$U\left(x\right)=\left[\begin{array}{ccc} q_{0}\cdots k_{0} & \cdots & q_{0}\;k_{h}\\ \vdots & \ddots & \vdots \\ q_{N}\;k_{0} & \cdots & q_{N}\;k_{h} \end{array}\right],$$

*x* is replaced by $F_{EFL}$.

The computational nature of CN shows that this is an *l_2_*-normalization, acting successively on the feature channels of *K_U_^T^V_U_* and *Q_U_*. Similarly, based on the analysis of the graph convolutional network [42] for the Laplace matrix *L = D − E* in place of the adjacency matrix *E*, the offset matrix can diminish the effect of noise [16]. This method provides sufficient discriminative feature information. Therefore, the offset method is also designed to efficiently learn the representation of the distinction of the embedded features. Additionally, the output feature is further obtained through an LBR network and an element-wise addition operation with the input feature.

As the output dimension of each layer is kept the same as the input features, the output of the single UFO attention layer is concatenated four times through the feature dimension, followed by a linear transformation, and more features are obtained. This process can be denoted as:

(13) $$F_{1}{=UT}^{1}{(F}_{EFL})$$

(14) $$F_{i}{=UT}^{i}{(F}_{i-1}),i=2,\;3,\;4,\;$$

(15) $$F_{o}=concat\left(F_{1}{,\;F}_{2}{,\;F}_{3}{,\;F}_{4}\right)\cdot W_{i},\;$$

where ${\;UT}^{i}$ represents the *i*th single UFO attention layer; $W_{i}\;$ is the weights of the linear layer.

Then, the input features and the output of stacked UFO attention layers are concatenated to fully obtain contextual features.

## 4. Experiments and Results

In this section, we first introduced the experimental settings as well as some general parameters and experimental data. Then, we showed how to train UFO-Net to perform the shape classification tasks. Immediately, we compared our model to other existing methods quantitatively and qualitatively. We evaluated the performance of the network on two public classification datasets. We implemented the project with Pytorch [43] and Python. This paper involved experiments using a single Tesla T4 GPU card under CUDA 10.0.

The overall framework of UFO-Net network is shown in Figure 1. The input point cloud contained only three-dimensional space coordinate information (x, y, z). The model derived 64-dimensional features from the embedding module and subsequently fed them to the transformer block. To examine the performance of our network, we replaced the two SG modules in PCT with two ASG and EFL modules and replaced the original attention mechanism with stacked UFO attention layers as the backbone. In particular, the number of nearest neighbors *k* for ASG was set as 32, derived from subsequent ablation experiments. To classify the input point cloud data into $N_{C}$ categories, the output processed by a max-pooling (MP) operator and an average-pooling (AP) operator were concatenated on the learned point-wise feature to obtain the global feature sufficiently. The decoder consisted of two cascaded feed-forward neural network LBRDs layers (including Linear, BatchNorm (BN), and LeakyRelu layers, each with a probability of 0.2 and a dropout rate of 0.5). The final classification score was predicted by a linear layer.

During training, to prevent overfitting, we performed random input dropout, random panning, and random anisotropic scaling operations to augment the input point clouds. The same soft cross-entropy loss function as [16] was adopted. The stochastic gradient descent (SGD) optimizer with a momentum of 0.9 and a weight decay of 0.0001 was used for training. During the testing period, a post-processing voting strategy was used. For 300 training phases, the batch size was set to 32, and the initial learning rate was 0.01, with a cosine annealing schedule to adjust the learning rate at each epoch. We chose the mean classification accuracy (mAcc) and the overall accuracy (OA) as evaluation metrics for the experiment.

### 4.1. Experiments on ModelNet40 Dataset

The ModelNet40 dataset is a widely used benchmark for point cloud shape classification proposed by Princeton University [43]. It contains 12,311 CAD models of 40 classes of man-made objects in the 3D world. For a fair comparison, we divided the dataset into a training/testing ratio of 8:2 following the convention, with 9843 universally divided objects for training and 2468 objects for testing. Using a common sampling strategy, each object was sampled uniformly to 1024 points and normalized to the unit length.

As shown in Table 1, we compare the proposed UFO-Net with a series of previous representative methods. The results of the classification experiments indicate that UFO-Net can effectively aggregate the global features of the point cloud. In Table 1, the mAcc and OA of the ModelNet40 dataset are 90.8% and 93.7%, respectively. As shown in Table 1, we can observe that (1) compared to the classical point-based PointNet, the mAcc of UFO-Net increased by 4.6%, and the OA improved by 4.5%. (2) Compared to the convolution-based DGCNN, the mAcc of UFO-Net increased by 0.6%, and the OA increased by 0.8%. (3) Compared to the transformer-based LFT-Net, the mAcc of UFO-Net increased by 1.1%, and the OA increased by 0.5%. We can also observe from Table 1 that almost all of the voxel-based methods perform worse than the point-based methods. Therefore, our method can effectively learn the spatial invariance of point clouds, and the network has obvious advantages over other methods in 3D object classification.

To further explore the neighbor feature extraction capability of our UFO-Net, we evaluate the accuracy of each class. The classification accuracy calculation results are shown in Table 2. When the model is tested, the data are classified according to the label. Models with the same label are grouped into the same category to create 40 model categories. The number in parentheses after each category indicates the number of models. Under a given number of test models, the classification accuracy of 10 objects—such as airplane, bed, bowl, guitar, laptop, person, sofa, stairs, stool, toilet—reaches 100%. Although there are also some categories that have a low classification accuracy, it can be seen that the classification accuracy rate of most categories is high. Therefore, it can be concluded that our model has good feature extraction ability for some objects that are important for edge articulation features.

### 4.2. Experiments on ScanObjectNN Dataset

Due to the rapid development of point cloud research, it can no longer fully meet some practical needs. For this reason, we also conducted experiments on the Scanned Object Neural Network dataset (ScanObjectNN) [53], a real-world point cloud dataset based on LiDAR scanning. ScanObjectNN is a more cumbersome set of point cloud category benchmarks, dividing about 15k objects in 700 specific scenarios into 15 classes and 2902 different object instances in the real world. The ScanObjectNN dataset has some variables, of which we are considering the most troublesome variable in the evaluation (PB_T50_RS). Each perturbation variable (prefix PB) in this dataset randomly shifts from the box centroid of the bounding box to 50% of the original size along a specific axis. Suffixes R and S represent rotation and scaling, respectively [53]. PB_T50_RS contains 13,698 real-world point cloud objects from 15 categories. In particular, 11,416 objects are used for training and 2282 objects are used for testing. This dataset is an especially large challenge for existing point cloud classification techniques. In this experiment, each point cloud object sampled 1024 points, and the model was trained using only the local (x, y, z) coordinates.

For real-world point cloud classification, we use the same network, training strategy, and 1000 3D coordinates as input. We quantitatively compared our UFO-Net with other state-of-the-art methods on the hardest ScanObjectNN benchmark dataset. In Table 3, we show the results of competing methods for scanning objective network datasets. Our network has an overall accuracy of 83.8% and an average class accuracy of 82.3%, which is a significant improvement on this benchmark. The results show that mAcc is improved by 5% and that OA is increased by 3.8% compared to the classical PCT. Furthermore, even when measured using the dynamic local geometry capture network RPNet++, we still have a fairly good lifting in mAcc and OA, with increments of 2.4% and 1.8%, respectively, which seems to be tailor-made for this dataset. Additionally, we observe that our UFO-Net creates the smallest gap between mAcc and OA. This phenomenon shows that our method has good robustness.

Since the ScanObjectNN dataset has some difficult cases to classify, the presence of feature-independent background points in ScanObjectNN can pose a challenge to the network. To obtain a global representation of the point cloud, we use the ASG module to learn a local fine-grained feature representation. This is because the design of ASG enhances the relationships between points and enriches the information of geometric features distributed on long edges. Furthermore, our approach provides an efficient solution with stacked UFO attention layers aiming to minimize the impact of these points by equally weighting them according to their channel affinity.

### 4.3. Model Complexity

We now compute the complexity of UFO-Net with previous state-of-the-art methods on ModelNet40 dataset [43], as shown in Table 4. We compared the number of model parameters to different creative algorithms. PointNet and PointNet++ have fewer parameters as they only use MLPs to extract features. Additionally, DGCNN and PCT also have few parameters, while KPConv and Point Transformer have more parameters due to their complex network designs. Despite this, our UFO-Net achieves a higher accuracy of 93.7%. Notably, our method achieves a similar parameter count to PointNet yet realizes state-of-the-art (SOTA) performance on ModelNet40. This result reveals that UFO-Net effectively improves attention-based methods.

## 5. Ablation Studies

To further investigate the effectiveness of our proposed method, we also conducted alternative comparative experiments on the ModelNet40 dataset. In order to ensure the fairness of the experiment, the details of experiments remained unchanged. We experienced a comprehensive empirical analysis of the ASG module and stacked UFO attention layers. A series of ablation experiments were reported to verify the effectiveness of the proposed module. In detail, we explored the impact of some important hyper-parameters, such as the impact of different point sampling densities, the number of nearest neighbors in ASG, and *h* parameter in stacked UFO attention layers.

### 5.1. Impact of Point Density

**Figure 5:**
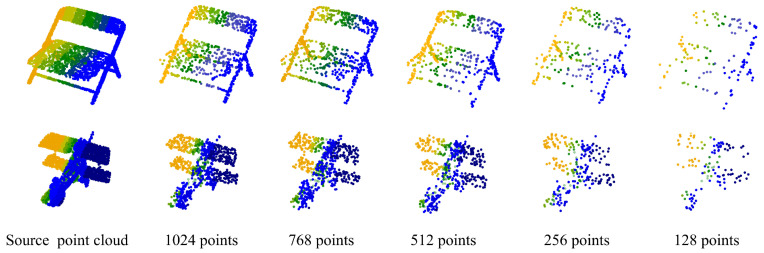
Examples with different point sampling densities on chair and airplane.

Robustness of point cloud density. Sampling density has an influence on point clouds, as shown in Figure 5. Therefore, we conducted experiments at different point densities to evaluate the performance of the proposed network.

**Figure 6:**
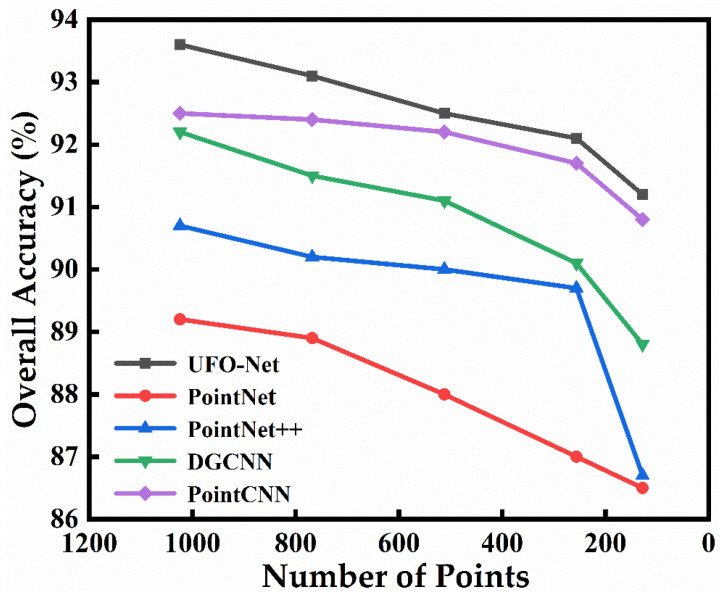
Classification results for different input points on the ModelNet40 dataset.

The accuracy of experimental curves for different point densities is shown in Figure 6. From Figure 6, it can be seen that the use of 1024 points to train the model to extract features is effective for the designed network. In real-world scenarios, however, point clouds are always fragmented and do not completely cover the surface of the target object. Therefore, in this section, we also conduct experiments at different point densities to evaluate the performance of our network. Figure 6 shows the overall accuracy at different input points on the ModelNet40 dataset. We trained our network using random samples of 1024, 768, 512, 256, and 128 input points. The curves in the figure expose the accuracy trend of the classification model test. Compared to other methods, our model has good robustness for point cloud density. Even at low point densities, our network maintains good accuracy. For example, 91.1% can be reached at 128 input points. The results show that this model can be widely used in different point densities. For relatively sparse scenes, the model can still work efficiently with contextual features.

### 5.2. Impact of h Parameter

To further explore the parameter setting details of the stacked UFO attention layers, we evaluated the effect of h on the model performance. For the ModelNet40 dataset, we fairly built a set of experiments corresponding to the value *h* in each single UFO attention layer. The experimental settings remain unchanged. The experimental results for different *h* values are shown in Table 5.

We sequentially evaluated the experimental results of the proposed network at 2, 4, 8, and 16 *h* values. In the original network, *h* was set to 4. We trained and tested all *h* parameters using the same dataset to perform fair quantitative evaluations. It can be seen from Table 5 that if *h* is 2, the final overall accuracy is reduced by 1.3%; if *h* is 8, the final overall accuracy is reduced by 0.8%; if *h* is 16, the final overall accuracy is reduced by 0.5%. Therefore, it can be concluded that the network performs best when the *h* parameter is set to 4. Lastly, there is no doubt that this method can achieve comparable results regardless of the value of *h*. The results further indicate good stability of our approach on the ModelNet40 dataset.

**Figure 7:**
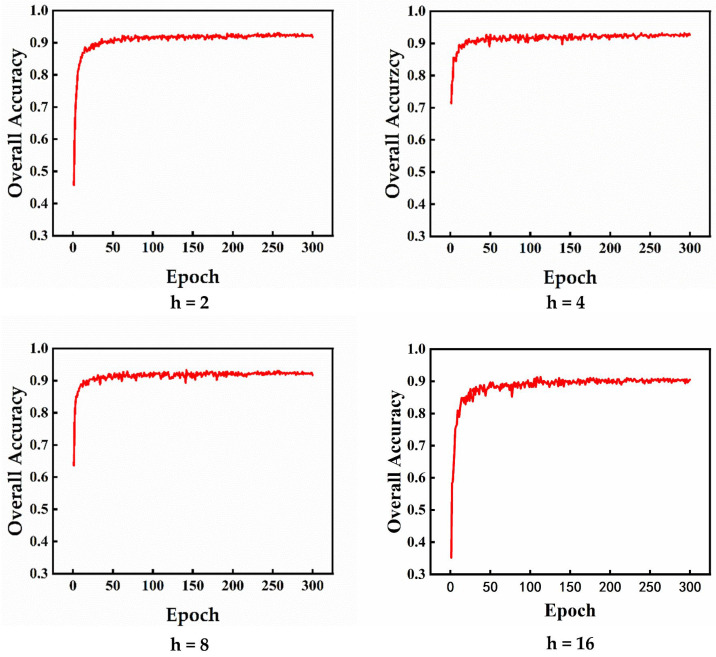
Four running results (OA) of UFO-Net with different *h* values on the ModelNet40 test dataset, in which *h* represents the value of UFO’s head.

Immediately after, we evaluated the testing OA curves for *h* values of 2, 4, 8, and 16 on the ModelNet40 dataset. The results are demonstrated in Figure 7. It can be seen from the figure that the model training climbs slowly when *h* = 2 or 16. When *h* is 4 or 8, the model can reach high accuracy rate quickly, and the former reach the fitness peak more quickly. Overall, it seems that all of our models have fast convergence ability.

### 5.3. The Impact of Querying Methods and Number of Neighbors

The proposed approach relies heavily on the ASG module. In addition to the above large number of experiments, the adaptability of the neighbor point selection method to the network is also studied. The mainstream range search methods for obtaining local features today are ball query and KNN search. The former ball method usually returns all points in the radius range class of a point [11], while the latter KNN returns a fixed number of points near a point [12]. We tested the impact of the two methods on UFO-Net separately. Here, to keep the experiment fair, we guaranteed that the number of neighbors was the same. The testing results on the ModelNet40 dataset are shown in Table 6. The results show that the proposed ASG method prefers the *k*-nearest neighbor selection method for the local feature aggregation. Therefore, it obtains more valuable feature information when acquiring features.

Additionally, we also evaluated the effect of the number of points in each neighborhood, which was the effect of the number *k*. In order to ensure the fairness of the experiment, four stacked UFO attention layers were the same in this experimental stage. Table 7 shows the results of the value of *k* on accuracy. In this experiment, we sampled some representative numbers of nearest neighbors to test UFO-Net. This paper reported results of 8, 16, 24, 32, 40, and 48, with *k* = 32 achieving the best results. From Table 7, if the value of *k* is small, the neighborhood characteristics cannot be fully expressed. This may be because strong feature correlation cannot be achieved between attention layers. Certainly, when the value of *k* is large, the Euclidean distance cannot estimate its geometry, which will produce a large noise deviation during feature extraction. Our network achieves the highest mAcc of 90.8% and OA of 93.7% when *k* is 32.

## 6. Conclusions

In this paper, we designed a new point cloud classification network called UFO-Net. A novel transformer method suitable for learning irregular domain point cloud is proposed. The linearly normalized attention mechanism is employed in point cloud processing tasks, as it can help to mitigate the effects of differences in scale between different features. In order to solve the problem of the uneven sampling of points in the local feature extraction module, we introduced a novel augmented sampling and grouping (ASG) module. This module reconsiders a local feature aggregation module and a more comprehensive method of feature processing. This model also employs an effective feature learning (EFL) pipeline connecting ASG and stacked UFO attention layers for processing geometric features. Among them, our module generates rich contextual information and is able to capture spatial features with significant local feature variations through stacked UFO attention layers. This method achieved state-of-the-art results in the task of classifying point clouds using only 3D coordinates as input. Moreover, ablation experiments show that our method achieves a better performance than other current frameworks. What’s more, we hope that this work will provide further research into the characteristics of transformers in point cloud processing tasks. Additionally, we plan to investigate the idea of the proposed architecture in part segmentation tasks and semantic segmentation tasks.

## Figures and Tables

**Table 1:** Comparison to state-of-the-art approaches on the ModelNet40 dataset.

Method	Representation	Input Size	mAcc (%)	OA (%)
3DShapeNets [44]	voxel	30^3^	77.3	84.7
VoxNet [20]	voxel	32^3^	83.0	85.9
MVCNN [45]	multi-view	12 × 224^2^	-	90.1
GVCNN [46]	multi-view	8×	-	93.1
ECC [24]	point	1024 × 3	83.2	87.4
PointNet [10]	point	1024 × 3	86.2	89.2
DeepNet [47]	point	5000 × 3	-	90.0
PointNet++ [11]	point+normal	1024 × 3	-	91.9
PointCNN [31]	point	1024 × 3	88.1	92.2
PCNN [48]	point	1024 × 3	-	92.3
GAPNet [29]	point	1024 × 3	89.7	92.4
DGCNN [12]	point	1024 × 3	90.2	92.9
KPConv [49]	point	7000 × 3	-	92.9
RS-CNN [40]	point+normal	1024 × 3	-	92.9
DRNet [50]	point	1024 × 3	-	93.1
MFNet [51]	point	1024 × 3	91.4	93.1
PCT [16]	point	1024 × 3	-	93.2
LFT-Net [52]	point+normal	1024 × 3	89.7	93.2
AGNet [25]	point	1024 × 3	90.7	93.4
Point Transformer [15]	point	1024 × 3	90.6	93.7
UFO-Net	point	1024 × 3	90.8	93.7

**Table 2:** Classification accuracy under different categories on ModelNet40.

Categories	Accuracy	Categories	Accuracy	Categories	Accuracy	Categories	Accuracy
airplane (100)	100%	cup (20)	65%	laptop (20)	100%	sofa (100)	100%
bathtub (20)	98%	curtain (20)	96%	mantel (100)	95%	stairs (20)	100%
bed (100)	100%	desk (86)	93%	monitor (100)	98%	stool (20)	100%
bench (20)	81%	door (20)	95%	nightstand (86)	62%	table (100)	91%
bookshelf (100)	98%	dresser (86)	89%	person (20)	100%	tent (20)	97%
bottle (100)	97%	flowerpot (20)	34%	piano (20)	95%	toilet (100)	100%
bowl (20)	100%	glass box (20)	90%	plant (100)	86%	tv stand (100)	87%
car (100)	99%	guitar (100)	100%	radio (20)	75%	vase (100)	86%
chair (100)	97%	keyboard (20)	95%	range hood (100)	95%	wardrobe (20)	81%
cone (20)	96%	lamp (20)	92%	sink (20)	96%	xbox (20)	91%

**Table 3:** Classification results on the ScanObjectNN dataset.

Method	mAcc (%)	OA (%)
3DmFV [9]	58.1	63.0
PointNet [10]	63.4	68.2
PointNet++ [11]	75.4	77.9
SpiderCNN [54]	69.8	73.7
DGCNN [12]	73.6	78.1
PointCNN [30]	75.1	78.5
BGA-DGCNN [53]	75.7	79.7
PCT [16]	77.3	80.0
DRNet [50]	78.0	80.3
GBNet [38]	77.8	80.5
RPNet++ [55]	79.9	82.0
UFO-Net	82.3	83.8

**Table 4:** Comparisons of UFO-Net’s complexity on the ModelNet40 dataset.

Method	Input	Parameters	OA (%)
PointNet [10]	1024	3.47 M	89.2
PointNet++ [11]	1024	1.48 M	91.9
KPConv [48]	1024	15.2 M	92.9
InterpCNN [56]	1024	12.5 M	93.0
DGCNN [12]	1024	1.81 M	92.9
Point Transformer [15]	1024	4.34 M	93.7
PCT [16]	1024	2.88 M	93.2
3DCTN [57]	1024	4.22 M	93.3
UFO-Net	1024	3.46 M	93.7

**Table 5:** Experimental results of *h* parameter setting on the ModelNet40 dataset.

Value of *h*	mAcc (%)	OA (%)
2	89.4	92.4
4	90.8	93.7
8	89.6	92.9
16	90.2	93.2

**Table 6:** Experimental results of different querying methods.

Grouping Method	Neighbors	OA (%)
ball query	32	93.1
KNN	32	93.7

**Table 7:** Experimental results of UFO-Net on ModelNet40 test set with *k* neighbors in the definition of ASG module.

Number of Neighbors (*k*)	mAcc (%)	OA (%)
8	86.4	89.4
16	87.6	91.2
24	89.6	92.1
32	90.8	93.7
40	89.9	92.5
48	88.6	91.8

## Data Availability

Not applicable.

## Abbreviations

The following abbreviations are used in this paper:

3D	Three-Dimensional
CNNs	Convolutional Neural Network
UFO	Unified Force Operation
KNN	K-Nearest Neighbor
GLNs	Grouped Local Neighbors
MLP	Multi-Layer Perceptrons
mAcc	Mean Classification Accuracy
LiDAR	Light Detection and Ranging
SA	Self-Attention
FPS	Farthest Point Sampling
ASG	Augmented Sampling and Grouping
EFL	Effective Feature Learning
ReLU	Rectified Linear Unit
OA	Overall Accuracy

## References

[1] Rahman M.M., Tan Y., Xue J., Lu K. (2020). Recent advances in 3D object detection in the era of deep neural networks: A survey. IEEE Trans. Image Process..

[2] Schwarz B. (2010). Lidar: Mapping the world in 3D. Nat. Photonics.

[3] Zhang R., Candra S.A., Vetter K., Zakhor A. Sensor Fusion for Semantic Segmentation of Urban Scenes. Proceedings of the 2015 IEEE International Conference on Robotics and Automation (ICRA).

[4] Dewi C., Chen R.C., Yu H., Jiang X. (2021). Robust detection method for improving small traffic sign recognition based on spatial pyramid pooling. J. Ambient. Intell. Humaniz. Comput..

[5] Biswas J., Veloso M. Depth Camera Based Indoor Mobile Robot Localization and Navigation. Proceedings of the IEEE International Conference on Robotics and Automation (ICRA).

[6] Zermas D., Izzat I., Papanikolopoulos N. Fast Segmentation of 3d Point Clouds: A Paradigm on Lidar Data for Autonomous Vehicle Applications. Proceedings of the IEEE International Conference on Robotics and Automation (ICRA).

[7] He K., Zhang X., Ren S., Sun J. (2016). Deep Residual Learning for Image Recognition. Proceedings of the 2016 IEEE Conference on Computer Vision and Pattern Recognition (CVPR).

[8] Zhao H., Jia J., Koltun V. Exploring self-attention for image recognition. Proceedings of the IEEE/CVF Conference on Computer Vision and Pattern Recognition (CVPR).

[9] Ben-Shabat Y., Lindenbaum M., Fischer A. (2018). 3DmFV: Three-dimensional point cloud classification in real-time using convolutional neural networks. IEEE Robot. Autom. Lett..

[10] Qi C.R., Su H., Mo K., Guibas L.J. PointNet: Deep Learning on Point Sets for 3d Classification and Segmentation. Proceedings of the IEEE Conference on Computer Vision and Pattern Recognition (CVPR).

[11] Qi C.R., Yi L., Su H., Guibas L.J. PointNet++: Deep Hierarchical Feature Learning on Point Sets in A Metric Space. Proceedings of the 31st International Conference on Neural Information Processing Systems (NIPS).

[12] Wang Y., Sun Y., Liu Z., Sarma S.E., Bronstein M.M., Solomon J.M. (2019). Dynamic graph CNN for learning on point clouds. ACM Trans. Graph..

[13] Park C., Jeong Y., Cho M., Park J. Fast point transformer. Proceedings of the IEEE/CVF Conference on Computer Vision and Pattern Recognition (CVPR).

[14] Han X.F., Jin Y.F., Cheng H.X., Xiao G.Q. (2022). Dual transformer for point cloud analysis. IEEE Trans. Multimed..

[15] Zhao H., Jiang L., Jia J., Torr P.H., Koltun V. Point transformer. Proceedings of the IEEE/CVF International Conference on Computer Vision (ICCV).

[16] Guo M.H., Cai J., Liu Z.N., Mu T.J., Martin R.R., Hu S. (2020). Pct: Point cloud transformer. Comput. Vis. Media.

[17] Vaswani A., Shazeer N., Parmar N., Uszkoreit J., Jones L., Gomez A.N., Kaiser Ł., Polosukhin I. Attention is all you need. Proceedings of the Advances in Neural Information Processing Systems (NeurIPS).

[18] Song J.G. (2021). UFO-ViT: High Performance Linear Vision Transformer without Softmax. arXiv.

[19] Le T., Duan Y. Pointgrid: A deep network for 3D shape understanding. Proceedings of the IEEE Conference on Computer Vision and Pattern Recognition (CVPR), Salt Lake City.

[20] Maturana D., Scherer S. (2015). VoxNet: A 3D Convolutional Neural Network for real-time object recognition. Proceedings of the IEEE/RSJ International Conference on Intelligent Robots and Systems (IROS).

[21] Vo A.V., Truong-Hong L., Laefer D.F., Bertolotto M. (2015). Octree-based region growing for point cloud segmentation. ISPRS J. Photogramm. Remote Sens..

[22] Klokov R., Lempitsky V. Escape from cells: Deep kd-networks for the recognition of 3d point cloud models. Proceedings of the 2017 IEEE International Conference on Computer Vision (ICCV).

[23] Meng H.Y., Gao L., Lai Y.K., Manocha D. Vv-net: Voxel vae net with group convolutions for point cloud segmentation. Proceedings of the IEEE/CVF International Conference on Computer Vision (ICCV).

[24] Simonovsky M., Komodakis N. Dynamic edge-conditioned filters in convolutional neural networks on graphs. Proceedings of the IEEE Conference on Computer Vision and Pattern Recognition (CVPR).

[25] Jing W., Zhang W., Li L., Di D., Chen G., Wang J. (2022). AGNet: An attention-based graph network for point cloud classification and segmentation. Remote Sens..

[26] Lin Z.H., Huang S.Y., Wang Y.C.F. Convolution in the cloud: Learning Deformable Kernels in 3D Graph Convolution Networks for Point Cloud Analysis. Proceedings of the IEEE/CVF Conference on Computer Vision and Pattern Recognition (CVPR).

[27] Lei H., Akhtar N., Mian A.S. (2020). Spherical kernel for efficient graph convolution on 3d point clouds. IEEE Trans. Pattern Anal. Mach. Intell..

[28] Li G., Müller M., Qian G., Perez I.C.D., Abualshour A., Thabet A.K., Ghanem B. (2023). DeepGCNs: Making GCNs go as deep as CNNs. IEEE Trans. Pattern Anal. Mach. Intell..

[29] Chen C., Fragonara L.Z., Tsourdos A. (2021). GAPNet: Graph attention based point neural network for exploiting local feature of point cloud. Neurocomputing.

[30] Xu M., Zhou Z., Qiao Y. Geometry Sharing Network for 3D Point Cloud Classification and Segmentation. Proceedings of the AAAI Conference on Artificial Intelligence (AAAI).

[31] Li Y., Bu R., Sun M., Wu W., Di X., Chen B. (2018). Pointcnn: Convolution on x-transformed points. Adv. Neural Inf. Process. Syst..

[32] Luong T., Pham H., Manning C.D. (2015). Effective Approaches to Attention-based Neural Machine Translation. arXiv.

[33] Bahdanau D., Cho K., Bengio Y. (2014). Neural Machine Translation by Jointly Learning to Align and Translate. arXiv.

[34] Tao Z., Zhu Y., Wei T., Lin S. (2021). Multi-head attentional point cloud classification and segmentation using strictly rotation invariant representations. IEEE Access.

[35] Wen X., Li T., Han Z., Liu Y.S. Point cloud completion by skip-attention network with hierarchical folding. Proceedings of the IEEE/CVF Conference on Computer Vision and Pattern Recognition (CVPR).

[36] Han X.F., He Z.Y., Chen J., Xiao G.Q. (2022). 3CROSSNet: Cross-level cross-scale cross-attention network for point cloud representation. IEEE Robot. Autom. Lett..

[37] Wang G., Zhai Q., Liu H. (2022). Cross self-attention network for 3D point cloud. Knowl. Based Syst..

[38] Qiu S., Anwar S., Barnes N. (2021). Geometric back-projection network for point cloud classification. IEEE Trans. Multimed..

[39] Zheng S., Pan J., Lu C.-T., Gupta G. (2022). PointNorm: Normalization is All You Need for Point Cloud Analysis. arXiv.

[40] Liu Y., Fan B., Xiang S., Pan C. Relation-shape convolutional neural network for point cloud analysis. Proceedings of the IEEE/CVF Conference on Computer Vision and Pattern Recognition (CVPR).

[41] Yu T., Meng J., Yuan J. Multi-view Harmonized Bilinear Network for 3D Object Recognition. Proceedings of the 2018 IEEE/CVF Conference on Computer Vision and Pattern Recognition (CVPR).

[42] Bruna J., Zaremba W., Szlam A., LeCun Y. Spectral networks and deep locally connected networks on graphs. Proceedings of the International Conference on Learning Representations.

[43] Paszke A., Gross S., Massa F., Lerer A., Bradbury J., Chanan G., Killeen T., Lin Z., Gimelshein N., Antiga L. Pytorch: An Imperative Style, High-Performance Deep Learning Library. Advances in Neural Information Processing Systems 32 (NeurIPS 2019). https://proceedings.neurips.cc/paper/2019/hash/bdbca288fee7f92f2bfa9f7012727740-Abstract.html.

[44] Wu Z., Song S., Khosla A., Yu F., Zhang L., Tang X., Xiao J. 3D shapenets: A deep representation for volumetric shapes. Proceedings of the IEEE Conference on Computer Vision and Pattern Recognition.

[45] Su H., Maji S., Kalogerakis E., Learned-Miller E. Multi-view convolutional neural networks for 3d shape recognition. Proceedings of the IEEE International Conference on Computer Vision (ICCV).

[46] Feng Y., Zhang Z., Zhao X., Ji R., Gao Y. (2018). GVCNN: Group-view convolutional neural networks for 3D shape recognition. Proceedings of the IEEE Conference on Computer Vision and Pattern Recognition.

[47] Ravanbakhsh S., Schneider J.G., Póczos B. (2016). Deep Learning with Sets and Point Clouds. arXiv.

[48] Atzmon M., Maron H., Lipman Y. (2018). Point convolutional neural networks by extension operators. ACM Trans. Graph..

[49] Thomas H., Qi C.R., Deschaud J.E., Marcotegui B., Goulette F., Guibas L.J. Kpconv: Flexible and deformable convolution for point clouds. Proceedings of the IEEE/CVF International Conference on Computer Vision (ICCV).

[50] Qiu S., Anwar S., Barnes N. Dense-resolution network for point cloud classification and segmentation. Proceedings of the IEEE/CVF Winter Conference on Applications of Computer Vision (WACV).

[51] Li Y., Lin Q., Zhang Z., Zhang L., Chen D., Shuang F. (2022). MFNet: Multi-level feature extraction and fusion network for large scale point cloud classification. Remote. Sens..

[52] Gao Y., Liu X., Li J., Fang Z., Jiang X., Huq K.M.S. (2023). LFT-Net: Local feature transformer network for point clouds analysis. IEEE Trans. Intell. Transp. Syst..

[53] Uy M.A., Pham Q.H., Hua B., Nguyen D., Yeung S. Revisiting Point Cloud Classification: A new benchmark dataset and classification model on real-world data. Proceedings of the IEEE/CVF International Conference on Computer Vision (ICCV).

[54] Xu Y., Fan T., Xu M., Zeng L., Qiao Y. Spidercnn: Deep learning on point sets with parameterized convolutional filters. Proceedings of the European Conference on Computer Vision (ECCV).

[55] Sheshappanavar S.V., Kambhamettu C. Dynamic Local Geometry Capture in 3D Point Cloud Classification. Proceedings of the IEEE 4th International Conference on Multimedia Information Processing and Retrieval (MIPR).

[56] Mao J., Wang X., Li H. Interpolated Convolutional Networks for 3d Point Cloud Understanding. Proceedings of the IEEE/C-VF International Conference on Computer Vision (ICCV).

[57] Lu D., Xie Q., Gao K., Xu L., Li J. (2022). 3DCTN: 3D convolution-transformer network for point cloud classification. IEEE Trans. Intell. Transp. Syst..

